# Bidirectional Roles of TRPV1 in a Latent Sensitization Model of Myofascial Low Back Pain

**DOI:** 10.1002/ejp.70255

**Published:** 2026-03-26

**Authors:** Vivian Blechschmidt, Bastian Schlickenrieder, Jonathan R. Husk, Ulrich Hoheisel, Handan Mörz, Rolf‐Detlef Treede, Wolfgang Greffrath

**Affiliations:** ^1^ Department of Neurophysiology, Mannheim Center for Translational Neuroscience, Medical Faculty Mannheim Heidelberg University Mannheim Germany; ^2^ Department of Psychiatry and Psychotherapy Central Institute of Mental Health, Heidelberg University Mannheim Germany

## Abstract

**Introduction:**

Chronic primary low back pain (cpLBP) is a global health concern with poorly understood pathomechanisms, potentially involving spinal sensitization. The capsaicin receptor TRPV1 plays a crucial role in sensitization across pain models. This study employed a rat model of cpLBP induced by two nerve growth factor (NGF) injections into lumbar muscles to explore TRPV1's roles in NGF‐induced spinal sensitization.

**Material and Methods:**

Male wildtype (WT) and TRPV1^−/−^ rats of both sexes (*N* = 10 each) underwent behavioural tests post NGF and vehicle injections. Tests included low back pressure pain thresholds (PPT), paw withdrawal thresholds (PWT), heat pain thresholds (HPT), and exploratory behaviour. Spinal TRPV1 expression after NGF injections was assessed in WT rats.

**Results:**

WT rats displayed latent local mechanical hypersensitivity after a single NGF injection, turning into manifest after the second, with presynaptic TRPV1 upregulation. TRPV1^−/−^ rats showed pronounced local mechanical hypersensitivity after a single NGF injection, increasing after the second NGF injection. Repeated NGF injections led to anxiety‐like behaviour and delayed remote mechanical hypersensitivity in both genotypes.

**Discussion:**

TRPV1 is upregulated during manifest sensitization but is not essential for developing local or remote mechanical hypersensitivity. TRPV1^−/−^ rats lacked early remission after a single NGF injection, suggesting TRPV1 may be involved in homeostatic maintenance rather than induction of spinal sensitization.

**Significance Statement:**

TRPV1 contributes to NGF‐induced sensitization but is not required for its development. These findings put TRPV1's potential as a therapeutic target for hyperalgesia into perspective and suggest a potential regulatory role in pain homeostasis.

## 1 Introduction

Chronic primary musculoskeletal pain of the low back (cpLBP), formerly labelled nonspecific LBP, is the most burdensome nonfatal condition globally (GBD 2016 Disease and Injury Incidence and Prevalence Collaborators. 2017; Treede et al. 2019). Recent evidence links cpLBP to neuroplastic changes, including sensitization of dorsal horn neurons innervating lumbar muscles and fascia. Repeated intramuscular injections of nerve growth factor (NGF) in animal models mimic these changes: an initial hypersensitivity after a single NGF injection subsides, leaving a silent but sensitized state. This latent sensitization (LS) is initiated by peripheral noxious input but maintained by central mechanisms, where hyperexcitability is kept silent by tonic inhibitory control (Corder et al. 2013; Gerum and Simonin 2022; Hucho and Levine 2007; Marvizon et al. 2015). In the NGF model, Hoheisel et al. (2013) showed that intramuscular NGF initiates LS, which remains silent until a second injection unmasks it as prolonged hypersensitivity (manifest sensitization, MS). This finding has since been replicated (Li et al. 2024; Reed et al. 2022; Syrett et al. 2022).

Early childhood immobilization stress can similarly induce LS (La Porta and Tappe‐Theodor 2020; Singaravelu et al. 2021), whereas exercise or manual therapy mitigates it (de Azambuja et al. 2018; Reed et al. 2020). Spinal glia contribute to sensitization (Li et al. 2024; Sessler et al. 2021; Zhang et al. 2017).

NGF‐sensitive, trkA‐positive neurons express pain‐related molecules, including transient receptor potential channels TRPV1, TRPA1, and TRPM3 (Caterina et al. 1997; Vandewauw et al. 2018). NGF increases TRPV1 expression (Donnerer et al. 2005; Xue et al. 2007) and membrane incorporation (Zhang et al. 2005), enhancing TRPV1‐mediated responses (Caterina et al. 2000; Shu and Mendell 1999). Anti‐NGF treatments reduce TRPV1 expression and hyperalgesia in inflammatory models (Cheng and Ji 2008; Ji et al. 2002). NGF‐induced heat hyperalgesia is absent after TRPV1 antagonism (Eskander et al. 2015; Mills et al. 2013) or in TRPV1‐deficient mice (Chuang et al. 2001).

Beyond heat pain, behavioural (Caterina et al. 2000; Eskander et al. 2015; Ota et al. 2013), pharmacological (Mills et al. 2013), and electrophysiological (McGaraughty et al. 2008) studies suggest TRPV1 contributes to mechanical hypersensitivity, probably via dorsal horn sensitization. TRPV1 antagonism prevented and reversed mechanical hypersensitivity (Mills et al. 2013). TRPV1‐positive fibre input is essential for spinal long‐term potentiation underlying mechanical hyperalgesia in humans (Henrich et al. 2015). Systemic NGF‐induced mechanical allodynia, unlike thermal hyperalgesia, is mediated by TRPV1 indirectly through spinal mechanisms (Eskander et al. 2015). In delayed‐onset muscle soreness, TRPV1^−/−^ mice did not develop mechanical hypersensitivity after a single NGF injection (Ota et al. 2013). However, whether LS and MS from repeated NGF exposure depend on TRPV1 remains unclear.

This study investigates TRPV1's roles in NGF‐induced spinal sensitization modelling cpLBP. We investigated whether repeated intramuscular NGF injections upregulate spinal TRPV1 and transferred the NGF model to TRPV1‐deficient rats to assess its role in LS and MS. We hypothesized that TRPV1 contributes to NGF‐induced sensitization, predicting reduced lumbar muscle hypersensitivity in TRPV1‐deficient rats. Remote hypersensitivity at the hind paw, a feature of chronic widespread pain, was assessed to explore TRPV1's roles in local and remote hypersensitivity.

## 2 Methods

### 2.1 Animals

In total, 76 rats were used for this study. Behavioural experiments (series 2) were performed on 30 adult Sprague–Dawley (SD) rats (aged 10 weeks at the beginning of the experiments): 10 wildtype (WT) male rats and 20 TRPV1^−/−^ rats of both sexes (male = ♂, female = ♀). The NGF‐induced cpLBP model was previously shown to work in both sexes, with females exhibiting a somewhat delayed onset of sensitization but more signs of widespread hypersensitivity (Syrett et al. 2022). To align with 3R principles, only male WT rats were used to confirm model stability, while both male and female TRPV1^−/−^ were included to assess roles of TRPV1. This reduces animal use but may limit understanding of WT female responses.

WT rats were obtained from Janvier. The SD‐Trpv1^tm1sage^ breeding pairs were commercially purchased (Envigo, Netherlands) and the animals were bred in the animal house of the Medical Faculty Mannheim. The animals were group housed on a 12‐h light cycle with food and water ad libitum. All animals were monitored daily for signs of distress, such as lethargy, water intake, weight gain, changes in grooming behaviour, scratch marks, and abnormal posture. Additionally, spinal cord tissue for immunohistochemical experiments (series 1) was extracted from the 10 ♂WT rats after completion of the behavioural experiments; an additional 30 WT SD rats were treated with either NGF or phosphate‐buffered saline (PBS, control) and spinal cords were extracted for TRPV1 expression at earlier time points. DRG neurons for calcium imaging were cultured from ♂TRPV1^−/−^ (*n* = 3) and ♂WT (*n* = 1) rats. Twelve male rats (♂WT and ♂TRPV1^−/−^, *n* = 6 each) were used for pilot experiments (data not shown).

The experimental procedure and the number of animals used were approved by the regional board Karlsruhe and carried out in accordance with the German law on the protection of animals, EU Directive 2010/63/EU, and with the ethical proposals of the International Association for the Study of Pain (Zimmermann 1983). For the complete ARRIVE Guidelines 2.0 Essential 10 checklist (Knopp et al. 2015; du Percie Sert et al. 2020), refer to Table 1.

**TABLE 1:** The ARRIVE Guidelines essential 10 checklist (du Percie Sert et al. 2020; cf. Knopp et al. 2015).

**FIGURE 1:**
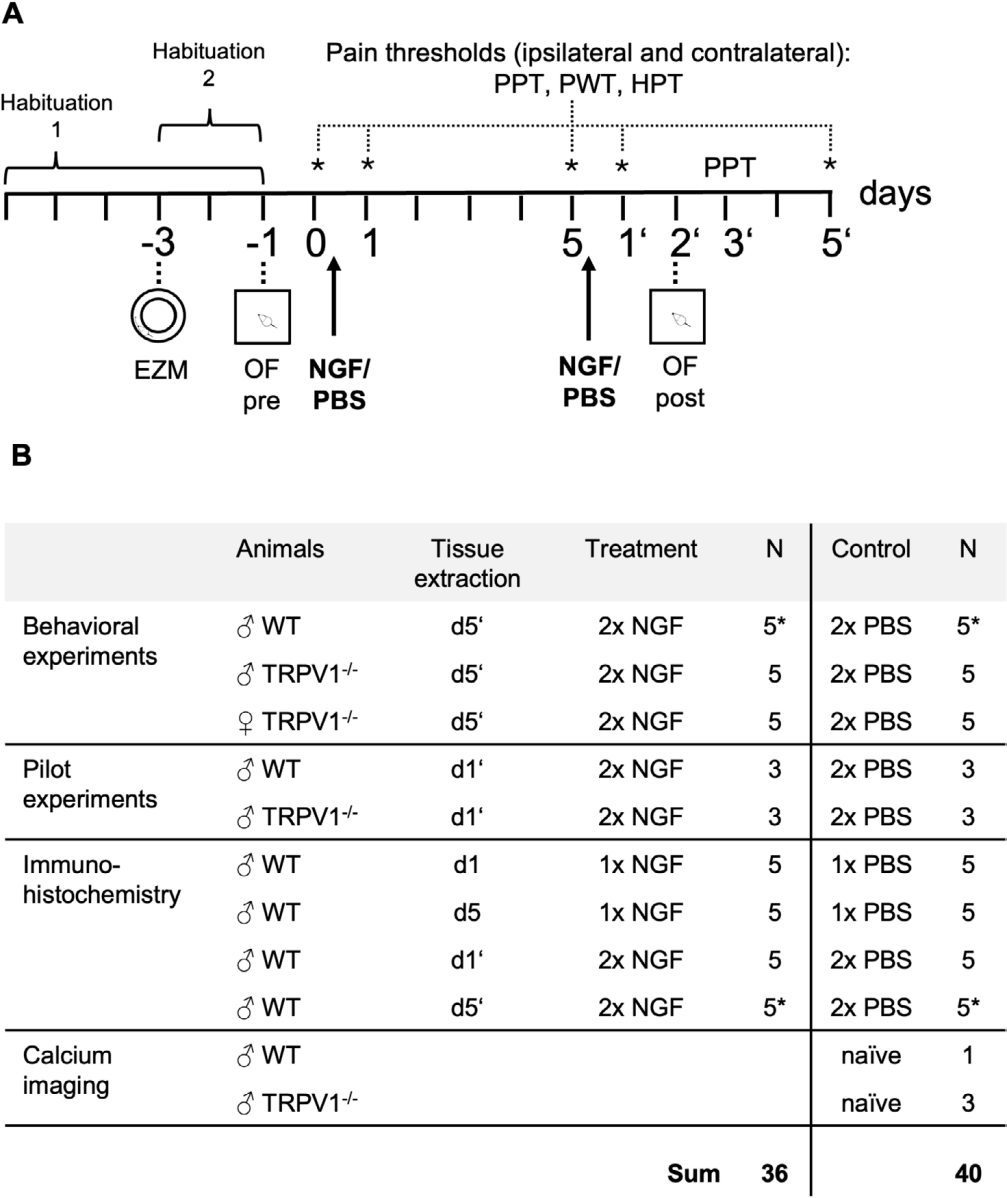
Experimental plan and group design. (A) Experimental plan of behavioural experiments. Habituation 1: Acclimatization to experimental room (60 min each), habituation 2: Acclimatization to experimental set up (grid, quartz plate; 10 min each). (B) Group design. Animals were treated with single i.m. injections (latent sensitization: 1× NGF vs. control (1× PBS)) or repeated injections (manifest sensitization: 2× NGF vs. 2× PBS). Time point of tissue extraction is shown in the respective column. * The animals marked with asterisks in the ‘Behavioural experiments’ and ‘Immunohistochemistry’ groups represent the same individuals. Tissue from the WT group in the d5′ condition was collected on d5′ following the completion of all behavioural tests. Time points shown are day 1 after the first injection (d1), day 5 after the first injection (d5), day 1 after the second injection (d1′), day 3 after the second injection (d3′) and day 5 after the second injection (d5′).

1. Study design
(a) Group design is given in Figure 1B and in the methods section (see “Animals”). The main experiments include six groups: Male WT NGF, male WT PBS, male TRPV1^−/−^ NGF, male TRPV1^−/−^ PBS, female TRPV1^−/−^ NGF, female TRPV1^−/−^ PBS. To minimize animal use, we compared male WT vs. TRPV1^−/−^ for genotype differences and male vs. female TRPV1^−/−^ for sex differences, omitting female WT vs. female TRPV1^−/−^ comparisons.
(b) The experimental unit is one animal. In figure 7, each dot represents one DRG neuron.
2. Sample size
(a) Number of animals in each treatment group are given in Figure 1B. The sample size and experimental procedures were approved and verified by the local ethics authority responsible for animal experimentation (regional board Karlsruhe, Germany), see section “Animals”.
(b) The sample size, based on Hoheisel et al. (2013), was 5 per group. Post hoc analysis showed a detectable effect size of *d* = 0.84, but only effects > 1.0 were highlighted for biological relevance, focusing on PPT values in male WT vs. TRPV1^−/−^ for NGF vs. PBS on Day 6 (see “Data Analysis”).
3. Inclusion and exclusion criteria
(a) Animals that fell off the elevated zero maze within less than 50% of the 5‐min recording period were excluded from analysis.
(b) Two WT rats and one male TRPV1^−/−^ rat were excluded from analysis due to falling from the EZM during the first 50% of the testing time.
(c) The exact number of animals (sample size for data evaluation) is given in the corresponding legend of each figure.
4. Randomization
(a) Randomization was used to allocate animals to treatment (NGF) or control (PBS) group. (b) To further minimize the danger of investigator bias, we strictly followed a standard protocol used in all treatment groups.
5. Blinding
The experimenters (V.B., B.S., J.R.H.) were blinded to the experimental conditions (NGF vs. PBS). U.Hor. held the unblinding code. Blinding in the elevated zero maze was unfeasible due to animal size differences, so automated exploratory analysis was conducted in EthoVision XT (Noldus) to assess genotype differences.
6. Outcome measures
(a) Outcome measures used to assess NGF model differences between wildtype and TRPV1^−/−^ rats include: pain thresholds: (1) pressure pain threshold of both multifidi muscles (g), (2) paw withdrawal threshold of both hind paws (g), (3) heat pain threshold assessed with graduated heat stimuli on the skin of the hind paws applied by a focused near‐infrared diode laser stimulator (mW); exploratory behaviour in the open field: (4) time spent in central zone (s), (5) time spent in corners (s)
(b) The primary outcome measure was the local pressure pain threshold of the injected multifidus muscle at the manifest sensitization stage (d6) in male rats.
7. Statistical methods
The statistical analysis is thoroughly outlined in the Methods section under “Data Analysis”, with specific tests noted in each figure legend. All statistical analyses were carried out with GraphPad Prism v.10. Cohen's *d* was manually calculated using Microsoft Excel.
8. Experimental animals
(a) Adult rats, outbred Sprague–Dawley, aged 10 weeks at experimental start, 220–460 g body weight, were used.
(b) All animals were monitored daily for signs of distress (e.g., lethargy, water intake, weight gain, changes in grooming behaviour, scratch marks, abnormal posture).
9. Experimental procedures
Experimental procedures are described in detail in the Methods section. Experimental details and time scale are also shown in Figure 1A. All procedures were done within the animal facility of the Medical Faculty Mannheim with approval of the local authorities (Regierungspräsidium Karlsruhe). The rationale for these procedures is outlined in the introduction and methods sections.
10. Results
Data are given as mean ± SEM; SEM is reported to indicate the precision of estimating mean values for statistical comparisons. Cohen's *d* > 1.0 (see “Data analysis”) is reported when appropriate.

### 2.2 Injection of Nerve Growth Factor Into the Multifidus Muscle

Injections of 50 μL NGF solution (human recombinant, Calbiochem Merck, Darmstadt, Germany; Hoheisel et al. 2013) at a concentration of 0.8 μM were made into the multifidus muscle 3 mm lateral of the spinous process L5. NGF was dissolved in PBS (pH 7.2–7.3). Two injections of NGF were given at an interval of 5 days unilaterally on the same side. Injections of 50 μL PBS solution served as control. About 50% of the animals showed nocifensive behaviour (withdrawal or escape behaviour) when applying the injection, however, regardless of whether PBS or NGF was given as previously described (Hoheisel et al. 2013).

### 2.3 Behavioural Tests

All 30 rats for behavioural testing arrived at the experimental building 2 weeks before the experiments started and were gradually accustomed to the experimental environment and examiners over 6 days before the first injection was given (Figure 1). All behavioural tests were performed between 8 am and 11 am during the light cycle. Rats were brought into the experimental room 1 h before behavioural testing began. Pilot studies were conducted to ensure that behavioural assays show consistency across multiple testing days in both naïve WT and TRPV1^−/−^ rats (*n* = 6 each). The investigators were blinded to the treatment condition when performing behavioural tests (B.S. & V.B. were blinded to experimental conditions (NGF vs. PBS), U.Hor. managed the unblinding code). However, blinding efforts for genotype were unsuccessful since age‐matched TRPV1^−/−^ rats were clearly smaller than ♂WT rats. To minimize the danger of bias, open field and elevated zero maze experiments which also aimed to determine genotype‐specific differences were analysed automatically with EthoVision XT8.5 tracking software (Noldus, Wageningen, Netherlands; Noldus et al. 2001). Where manual analysis of exploratory behaviour was necessary, analysis was performed via a standardized protocol by a third person that was not aware of the direction of weight differences between the animals of different genotypes (blinded investigator: J.R.H.; managing unblinding code: V.B.).

Behavioural tests included stimulation of both the ipsilateral and contralateral sides to ensure identical handling and to avoid differences related to unequal manipulation. Contralateral measurements were obtained but were not part of any of the predefined research questions and could therefore be considered descriptive only. Contralateral data are shown in Figure S1, Tables S1 and S2.

### 2.4 Elevated Zero Maze Test

To investigate behavioural differences between genotypes (WT vs. TRPV1^−/−^), anxiety‐related behaviour was tested using the elevated zero maze (EZM) test prior to any intervention. Rats were placed individually on an annular runway (100 cm diameter, 10 cm width, 80 cm height, matt black surface; made of thermoplastics) divided equally in four quadrants: two opposite zones enclosed by walls (27 cm high) and two open zones without any path boundary. The set up was illuminated by white light providing 50 lx in the open quadrants and 17–25 lx in closed quadrants. Rats were positioned with the head in the enclosed quadrant and the animal's behaviour was recorded for 5 min for analysis. The frequency and duration of entries into the open zone, number of transitions (complete crossing of an open zone), the latency to the first entry into the open zone and first transition, were analysed both automatically by EthoVision tracking software and manually by a person blinded to the genotype of the animal (blinded investigator: J.R.H.; managing unblinding code: V.B.). Blinding was challenging due to genotype‐specific weight differences, prompting us to seek an automated and objective method for data analysis. However, since the automated analysis using EthoVision for elevated zero maze (EZM) experiments has not been fully validated, we employed both manual and automated approaches. The results section highlights the parameters derived from automated analysis whenever they strongly correlated with manually analysed data (Spearman's rho > 0.9). Animals that fell off the EZM after less than 50% of the five‐minute recording period were excluded from analysis. Data from animals that fell off the EZM after more than 2.5 min recording time were extrapolated to 5 min and included in analysis. The EZM apparatus was cleaned with 70% ethanol before and after testing each rat.

### 2.5 Open Field Test

The locomotor activity of the animals was investigated through their exploratory behaviour in an unstructured open arena (100 cm × 100 cm, 50 cm high walls, wooden, matt black surface, illumination: 60 lx (corners) – 80 lx (center)). Rats were placed in the center of the open field and their movement was recorded for 10 min. The test was performed 1 day before the first injection (pretreatment) and 7 days after the first injection (posttreatment). Moving distance, velocity and immobility time were digitally recorded, analysed with EthoVision video‐tracking system, and reported as the total distance travelled (m), mean velocity (cm/s) and percentage of time in movement (%). The mean velocity was calculated from the locomotion velocity above 0 cm/s. Thigmotaxis, the tendency to remain close to walls, was assessed by measuring the time spent in the central zone (s) and time spent in the corners of the arena (s). The open field was cleaned with 70% ethanol before and after each test.

### 2.6 Mechanical Pain Threshold Testing

#### 2.6.1 Local Pressure Pain Threshold (PPT)

The mechanical pain threshold of the lumbar multifidus (MF) muscle was measured bilaterally at the vertebral level L5 through the intact skin with an electronic von Frey aesthesiometer (Life Science Instruments, Woodland Hills, CA, USA). A blunt, hemispherical tip with a diameter of 3.5 mm was used to determine the pain threshold of the loosely fixed animal with continuously increasing pressure stimulus intensity. This blunt sample—in contrast to pointed or angular samples—activates mainly nociceptors in the deep‐lying tissues such as the back muscles and fascia. Nociceptors in the overlying skin are only marginally activated, even at high pressure (Chacur et al. 2009; Kosek et al. 1999). The PPT was defined as the minimum of pressure intensity required to elicit a nocifensive reaction (withdrawal and escape behaviour, vocalization).

#### 2.6.2 Remote Paw Withdrawal Threshold (PWT)

To test for mechanical hypersensitivity at remote anatomical sites, mechanical hypersensitivity was tested bilaterally on both hind paws using an electronic von Frey aesthesiometer (Life Science Instruments, Woodland Hills, CA, USA). Rats were acclimatized for 15 min prior to testing on a mesh platform in clear Plexiglass animal enclosures. A sharp tip with a diameter of 0.8 mm was used to apply pressure with steadily increasing intensity on the intact skin until the first pain‐related reaction (withdrawal, vocalization) occurred which was defined as the pain threshold. This test was repeated three times on both hind paws with a 1 min break between each stimulus and the results are reported as mean PWT (g).

### 2.7 Confirmation of Genetic Ablation of TRPV1

Although only homozygous TRPV1^−/−^ animals were used for mating and, consequently, the TRPV1 receptor was supposed to be completely absent in all animals, we tested functional consequences of genetic ablation in two different ways: calcium imaging and behavioural testing.

Calcium imaging experiments of cultured DRG neurons (1 ♂WT rat, 3 ♂TRPV1^−/−^ rats, random sample) were performed according to a previously published protocol (Rosenberger et al. 2020). Capsaicin and resiniferatoxin (RTX), both potent TRPV1 agonists, were used to assess TRPV1‐specific responses. Capsaicin (M2028, Sigma) was dissolved in ethanol (100 mM) and RTX (R8756, Sigma) in DMSO (10 mM) as stock solution and stored at 4°C. Both substances were dissolved in Tyrode's solution (pH = 7.3) to their final concentration (capsaicin: 10 μM; RTX: 1 μM) just before each experiment. Potassium (140 mM KCl) was used to test for viability and excitability of the DRG neurons. None of these substrates changed the pH of the solutions by more than 0.1 and the maximum ethanol concentration was 0.1‰. Analysis of ratios of fluorescence intensity at 340 nm/380 nm excitation and subtraction of background fluorescence were carried out with xcellenceRT software (Olympus). Relative change of fluorescence ratio was calculated by dividing the peak value by the preceding baseline (mean fluorescence value within 1 min before application of the test substance). An increase of ≥ 23% was regarded as a significant response (Greffrath et al. 2001). DRG neurons were considered TRPV1‐positive when responding to capsaicin or RTX. Proportion of TRPV1‐positive cells to total viable neurons (= proportion of capsaicin‐ or potassium‐responsive cells) were compared between TRPV1^−/−^ and WT rats.

The heat pain threshold (HPT) was determined by application of graded heat stimuli on the skin of the hind paws with a focused diode laser stimulator of 1475 nm (diameter 10 μm, SK9‐2001, Schäfter + Kirchhoff, Hamburg, Germany). Laser‐induced heat stimulation enables non‐contact, purely thermal stimulation without accompanying effects from touch‐sensitive receptors. The duration of each laser‐induced heat stimulus was 200 ms. This temperature is perceived by test persons as moderately painful and does not leave any tissue damage (Rosenberger et al. 2020). Rats were acclimatized for 15 min prior to testing on a quartz glass platform in clear plexiglass animal enclosures. Test stimuli of increasing intensity were applied to both plantar hind paws. HPT were defined as the minimum stimulation intensity inducing nocifensive behaviour (paw licking, withdrawal or escape behaviour, vocalization) determined by the method of limits. Due to technical defects, thermal pain thresholds could not be determined in all animals (♂WT: *N* = 9, ♂TRPV1^−/−^: *N* = 7, ♀TRPV1^−/−^: *N* = 10).

### 2.8 Perfusion Fixation, Immunohistochemistry and Image Analysis

To determine TRPV1 expression after NGF injections, 40 adult ♂WT SD rats received either one or two injections of NGF or PBS (see 2.4 Injection of NGF). Spinal cord segment L2 was extracted after one or 5 days after a single i.m. injection (d1, d5; *n* = 5 each) or after two injections (d1′, d5′; *n* = 5 each). For the expression analysis of TRPV1, rats were transcardially perfused with 4% paraformaldehyde fixative solution. Spinal cord segments were extracted and cryopreserved in first 10% sucrose diluted in 0.1 M PBS for at least 24 h at 4°C and then 30% sucrose solution for another 24 h. 20 μm spinal cord cryosections were washed with PBS thrice for 5 min and then blocked with 10% Roti‐block. The sections were incubated with the primary antibody diluted in 1% Roti‐block solution overnight at 4°C.

TRPV1 (rabbit, 1:500; Cat. No. ACC‐030, Alomone Labs, Jerusalem, Israel) and NeuN (guinea pig, 1:1000; Cat. No. 266004, Synaptic Systems, Göttingen, Germany) primary antibodies were used to determine TRPV1 expression. The sections were washed in PBS (three 5‐min washes) and incubated with the secondary antibodies (donkey anti‐rabbit Cy3‐conjugated, 1:500, Cat. No. 711‐166‐152; donkey anti‐guinea pig Alexa Fluor 488, 1:500, Cat. No. 706‐546‐148, both Jackson Laboratory ImmunoResearch, Ely, UK) dissolved in 1% Roti‐block for 120 min at room temperature. Stained sections were imaged by using an upright fluorescent microscope (AXIO Imager.Z1, Zeiss, Oberkochen, Germany) using identical illumination exposure parameters for sections prepared from control and NGF treated rats, with parameters optimized for NGF‐treated tissue; thus, basal TRPV1 expression in PBS controls may appear faint. To exclude unspecific staining, negative control experiments were performed by omission of two primary antibodies (data not shown). For the quantification of signal intensities of TRPV1 immunoreactivity, we imaged and quantified 3 sections per segment. The signal intensity was quantified using FIJI/ImageJ software (National Institutes of Health, Maryland, USA; Schindelin et al. 2012). We measured the intensity exceeding default threshold. We qualitatively assessed location of spinal TRPV1 expression by 3D reconstruction in IMARIS 9.5 (Bitplane, Oxford Instruments, Wiesbaden, Germany) of three‐dimensional images acquired over a 20 μm *z*‐axis with a 1 μm step size with a laser scanning microscope (Leica DS SP5 equipped with CX PL APO CS 40.0x1.30 OIL UV; data not shown). Contralateral images were obtained but were not part of the predefined research questions and are therefore descriptive. To maintain a focused, hypothesis‐driven presentation, these data are not included in the Results or Discussion and are instead provided in (Figure S2).

### 2.9 Data Analysis

Animals were randomized into groups and examiners were blinded to experimental conditions (B.S. & V.B. were blinded to experimental conditions (NGF vs. PBS), U.Hor. managed the unblinding code). Minimal sample sizes for behavioural studies were based upon previous studies (Hoheisel et al. 2013; Sessler et al. 2021; Zhang et al. 2017). Post hoc power analysis determined that, with 5 subjects per group, the minimum detectable effect size was Cohen's *d* = 0.84, but only effect sizes greater than 1.0 were highlighted in the figures to focus on biologically meaningful differences. The calculation was based on the primary endpoint of local PPT values in multifidus muscles in male wildtype rats vs. TRPV1^−/−^ for NGF vs. PBS treatment on day 6 (manifest sensitization), using data from male wildtype rats in Hoheisel et al. (2013).

Behavioural comparisons by genotype (♂WT vs. ♂TRPV1^−/−^) and sex (♂ vs. ♀TRPV1^−/−^) were analysed using a *t*‐test for normally distributed data (Shapiro–Wilk test) or the Mann–Whitney *U*‐test for not normally distributed data.

Repeated measures ANOVA compared pain thresholds in NGF‐ and PBS‐treated animals across time points, using Geisser–Greenhouse correction. Normality (Shapiro–Wilk) and homogeneity of variance (Levene's) were tested beforehand. When assumptions were met, two‐way ANOVA with Tukey's post hoc for multiple comparisons was used. For violations, data were log‐transformed; if issues persisted, pairwise Welch's *t*‐tests were performed at each time point.

We used repeated measures ANOVAs with Geisser–Greenhouse correction and post hoc Dunnett's tests for multiple comparisons to analyse interactions of treatment (NGF vs. PBS), genotype (WT vs. TRPV1^−/−^), time point (d5 vs. d5′), and sex (male vs. female) on behavioural responses (PPT, PWT). We chose two different time points: d5 (latent sensitization) and d5′ (manifest sensitization). Two separate two‐way ANOVA analyses were performed to test for interactions between genotype and treatment, as well as sex and treatment. For HPT we used mixed effect model instead due to missing values.

Analyses used GraphPad Prism version 10.0 software reporting data as mean ± standard error of mean. Statistical significance was defined as *p <* 0.05. To describe effect sizes, Cohen's *d* was calculated for all pairwise comparisons. For normally distributed data, *d* was calculated as the mean difference divided by the pooled standard deviation. For non‐normal data, *η* ^2^ was first calculated and, where appropriate, transformed to d using *d* = $\sqrt{\eta^{2}}/\left(1-\eta^{2}\right)$ (Lenhard and Lenhard 2022). We defined *d* > 1.0 as a large effect, as this exceeded the detectable effect size from our post hoc power analysis (*d* = 0.84) and was chosen to highlight biologically relevant differences. Large effect sizes were interpreted independently of statistical significance to reflect the magnitude of observed differences, particularly in small‐sample mechanistic studies where power limitations may mask meaningful biological effects. However, *p*‐values were always reported alongside effect sizes, and when *p* ≥ 0.05, such results are described explicitly as non‐significant large effect sizes (Borenstein 1997; Giner‐Sorolla et al. 2024).

## 3 Results

### 3.1 Upregulation of TRPV1 in Superficial Dorsal Horn After One or Two NGF Injections

**FIGURE 2:**
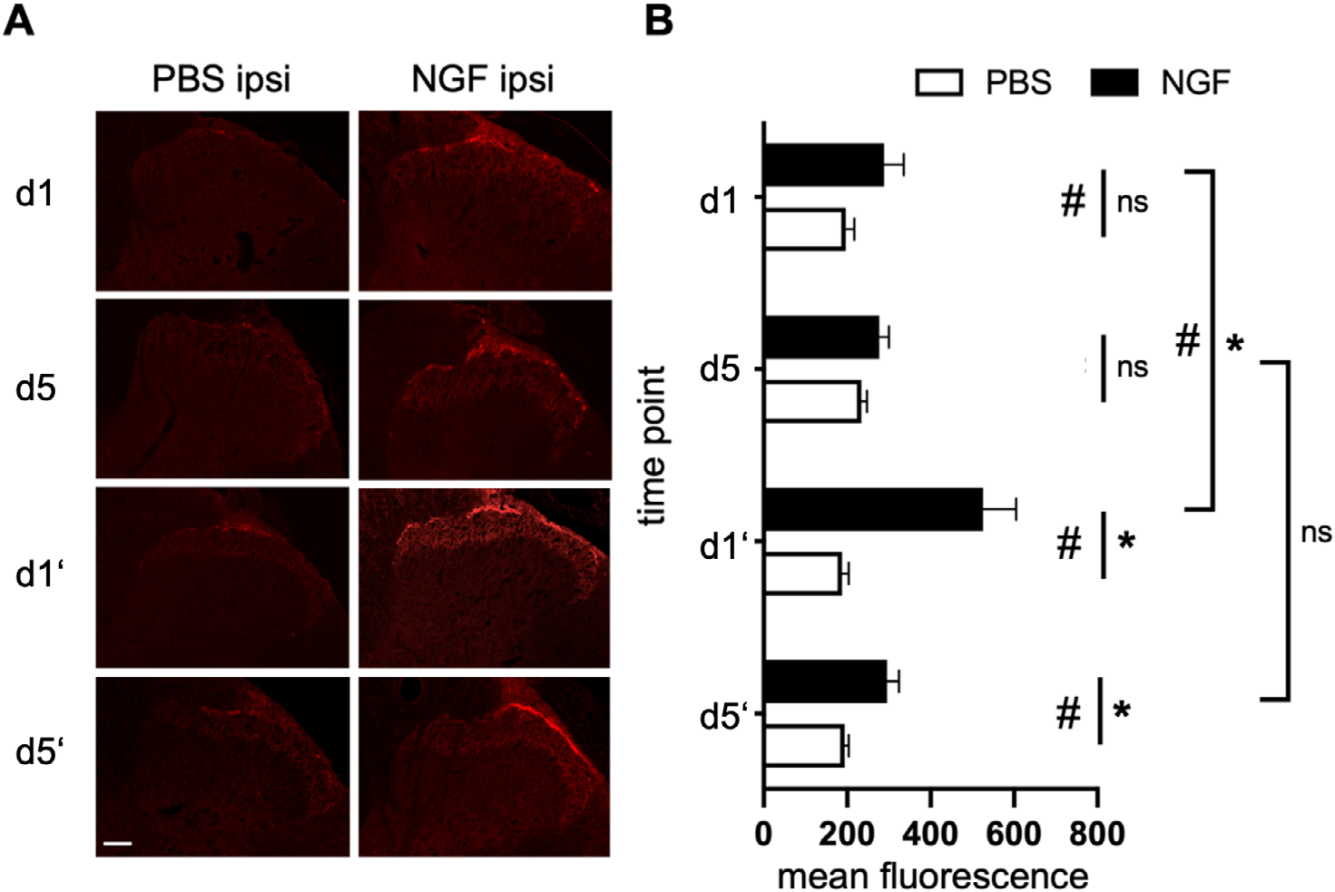
Repeated NGF injections enhance presynaptic TRPV1 expression in superficial laminae of the ipsilateral lumbar spinal cord. (A) Representative image of the mean fluorescence intensity of TRPV1 in the ipsilateral dorsal horn of the lumbar spinal cord segment L2. Images were acquired under identical conditions, with parameters optimized for NGF‐treated tissue; thus, basal TRPV1 expression in PBS controls may appear faint. (B) TRPV1 is time‐dependently upregulated in spinal dorsal horn after NGF injection (16 bit, scale: 0–65536). Mean ± SEM, *n* = 5 each, two‐way ANOVA. ns = not significant, *p* ≥ 0.05; * *p* < 0.05; # strong effect, Cohen's *d* ≥ 1. Scale bar: 200 μm. Time points shown are day 1 after the first injection (d1), day 5 after the first injection (d5), day 1 after the second injection (d1′), and day 5 after the second injection (d5′).

To study the contribution of TRPV1 in the NGF‐cpLBP model in ♂WT rats, a time‐course expression analysis of TRPV1 in the ipsilateral lumbar spinal cord segment L2 was performed at different time points after NGF/PBS injection (d1 and d5 after a single injection; d1′ and d5′ after a second injection, Figure 2, Table 2). We found a large but non‐significant increase of TRPV1 immunoreactivity 1 day after a single NGF injection (*p* = 0.13, *d* = 1.36) that had partly recovered by day 5 (*p* = 0.15, *d* = 0.98, Figure 2B). A subsequent second NGF injection led to a significantly larger increase (*p* = 0.01, *d* = 2.64) that was still substantial 5 days after the injection (*p* = 0.02, *d* = 2.15). We assumed that TRPV1 upregulation in the spinal segment L2 mainly occurred in primary afferents since the fluorescence signal was limited to Rexed laminae I/II where primary afferents of mainly C‐ and Aδ‐fibres terminate. Contralateral data are shown in Figure S2.

**TABLE 2:** Summary of statistical outcomes for behavioural and molecular parameters in NGF‐ and PBS‐treated rats.

	Baseline	After first injection		After second injection		
	d0	d1	d5	d1′	d3′	d5′
PPT ipsilateral						
NGF vs. PBS						
♂ WT	0.415/0.57	0.451/0.53	0.370/0.63	**0.002/3.43**	**0.002/3.47**	**0.008/2.42**
♂ TRPV1^−^/^−^	0.595/0.35	0.130/**1.19**	**0.005/2.54**	**< 0.001/4.40**	**< 0.001/7.38**	**< 0.001/4.74**
♀ TRPV1^−^/^−^	0.233/0.84	0.085/**1.29**	0.068/**1.38**	**0.029/1.68**	**0.007/2.36**	**0.001/3.34**
		d1–d0	d5–d0	d1′–d0	d3′‐d0	d5′‐d0
Within NGF group comparison vs. baseline						
♂ WT		**0.003/3.05**	0.919/0.07	**< 0.001/4.93**	**< 0.001/4.41**	**< 0.001/4.29**
♂ TRPV1^−^/^−^		**0.043/1.55**	**0.012/2.05**	**< 0.001/5.01**	**< 0.001/4.64**	**< 0.001/4.95**
♀ TRPV1^−^/^−^		0.523/0.42	0.434/0.52	0.107/**1.13**	0.056/**1.61**	**0.004/2.54**
	d0	d1	d5	d1′	d3′	d5′
PWT ipsilateral						
NGF vs. PBS						
♂ WT	0.841/0.14	0.719/0.29	0.915/0.04	0.426/0.53	Not tested	0.056/**1.50**
♂ TRPV1^−^/^−^	0.767/0.26	0.501/0.37	0.635/0.32	0.713/0.24	Not tested	0.067**/1.34**
♀ TRPV1^−^/^−^	0.593/0.30	0.543/0.48	0.767/0.29	0.207/0.86	Not tested	**0.003/2.73**
	d0	d1	d5	d1′	d3′	d5′
HPT ipsilateral						
NGF vs. PBS						
♂ WT	0.544/0.48	0.503/0.46	0.722/0.26	0.502/0.48	Not tested	0.067/**1.66**
♂ TRPV1^−^/^−^	0.391/0.69	0.655/0.35	0.929/0.07	0.608/0.40	Not tested	0.903/0.11
♀ TRPV1^−^/^−^	0.138/0.99	0.237/0.83	0.952/0.04	0.313/0.68	Not tested	0.210/0.87
	d0	d1	d5	d1′	d3′	d5′
Spinal TRPV1 expression						
NGF vs. PBS						
♂ WT	Not tested	0.126/**1.36**	0.147/0.98	**0.012/2.64**	Not tested	**0.020/2.15**

*Note:* Values indicate *p*‐value/Cohen's *d* for comparisons between NGF‐ and PBS‐treated groups and for within‐group comparisons versus baseline in NGF‐treated animals. Bold values in the table denote significant results (*p* < 0.05) or large effect sizes (*d* > 1.0). Effect sizes (Cohen's *d*) quantify the magnitude of differences irrespective of significance. Time points shown: baseline (d0), 24 h (d1) and 5 days (d5) after the first injection, and 24 h (d1′), 3 days (d3′), and 5 days (d5′) after the second injection.

### 3.2 Functional TRPV1 Expression in WT and TRPV1 ^−/−^ Rats

**FIGURE 3:**
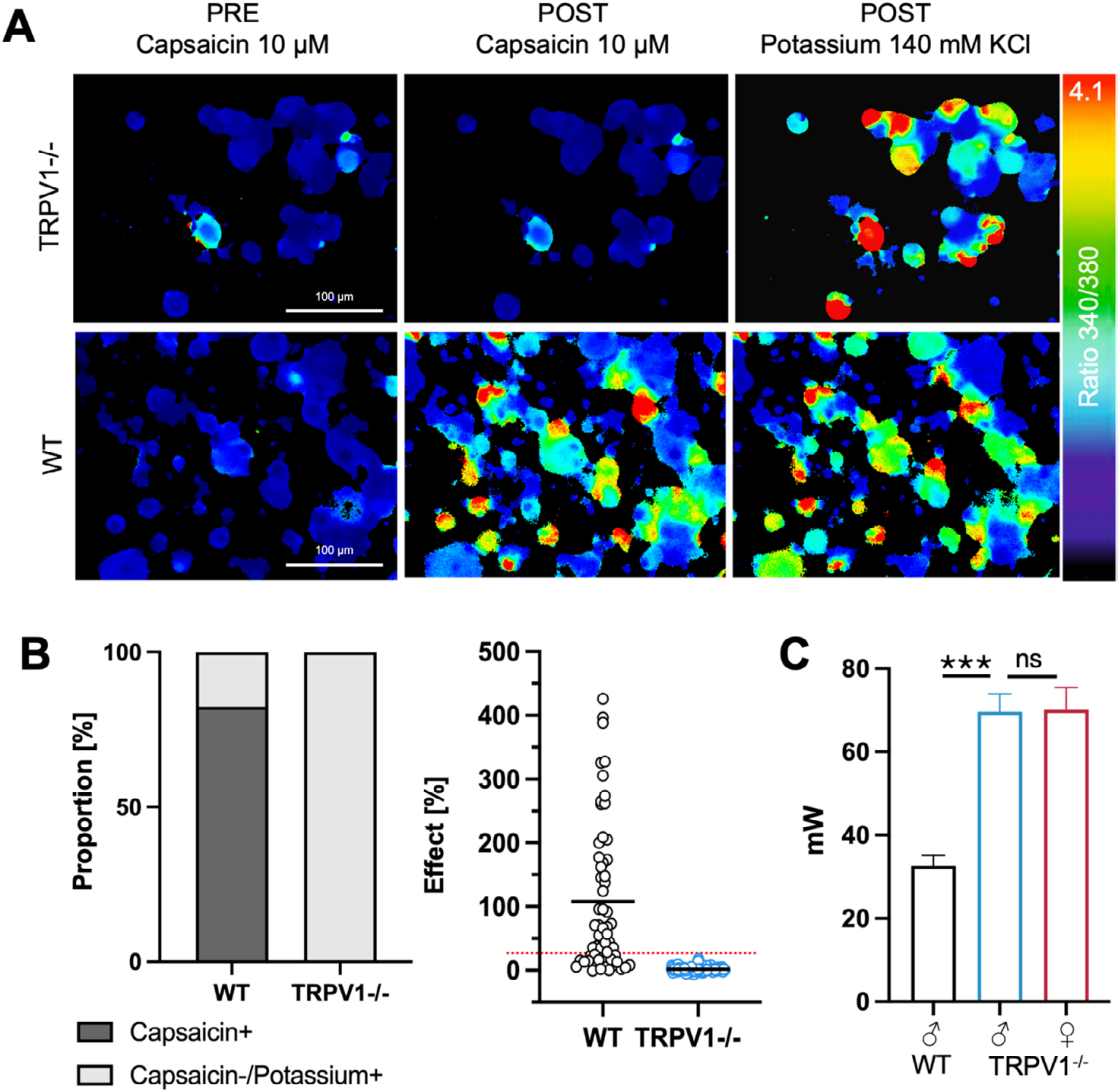
Absence of capsaicin‐responsive DRG neurons in TRPV1^−/−^ rats. (A) Example of false colour‐coded ratiometric calcium imaging pictures exhibiting the change in intracellular calcium in response to chemical stimulation (capsaicin, potassium): Upper row: TRPV1^−/−^ rat, lower row: WT rat. Scale bar: 100 μm. (B) Calcium responses of DRG neurons after capsaicin application. Left: Proportion of capsaicin‐responsive DRG neurons vs capsaicin‐unresponsive but potassium‐responsive neurons; middle: Relative change of intracellular calcium in response to capsaicin application; black line: Mean; dotted red line: 23%, limit above which a calcium increase was considered to be a response to capsaicin. (C) TRPV1^−/−^ rats required higher laser intensities (mW) to evoke nocifensive responses compared to WT, regardless of sex. Thresholds were calculated as the mean of ipsilateral and contralateral sides. Data are presented as mean ± SEM. Pairwise comparisons were made between male WT vs. male TRPV1^−/−^, and male vs. female TRPV1^−/−^, *n* = 7–10. Tests included t‐tests for normally distributed data or Mann–Whitney *U*‐test for non‐normal data, ns = not significant, *p* ≥ 0.05; ***p < 0.001.

To confirm the functional ablation of TRPV1, calcium‐imaging experiments with TRPV1 agonists (capsaicin, RTX) on cultured DRG neurons from three ♂TRPV1^−/−^ rats were performed. To ensure that with this setup capsaicin responses can be detected, calcium imaging experiments of DRG neurons of one ♂WT rat were performed. None of the 126 DRG neurons from TRPV1^−/−^ rats that exhibited a positive response to potassium but no response to vehicle (ethanol) responded to capsaicin or RTX (Figure 3A,B). In the WT animal, 82% of the excitable (potassium‐responsive) DRG neurons responded to capsaicin (Figure 3A,B), which is in line with previous literature (Greffrath et al. 2001; Wang et al. 2022).

Laser evoked heat pain thresholds (HPT) of both hind paws were significantly higher in ♂TRPV1^−/−^ rats compared with WT rats (HPT: *p* < 0.01, *d* = 3.9; Figure 3C, Table 2). In TRPV1^−/−^ rats, laser‐evoked pain is not lost completely because in the absence of TRPV1 other signal pathways can mediate heat detection (e.g., TRPA1, TRPM3; Vandewauw et al. 2018).

### 3.3 Body Weight and Anxiety‐Like Behaviour at Baseline

#### 3.3.1 Body Weight

While the animals were age matched (aged 10 weeks at the beginning of the experiments), they exhibited differences in body weight. WT rats were significantly heavier than ♂TRPV1^−/−^ rats (*p* < 0.01). Lower body weight is known for this strain (https://insights.envigo.com/hubfs/resources/data‐sheets/envigo‐gems‐trpv‐korat.pdf). Monitoring weight at various time points revealed comparable relative weight gain in wildtype and TRPV1^−/−^ rats (mean relative change d5′ – baseline: ♂WT 7.0%, ♂TRPV1^−/−^ 6.5%; ♀TRPV1^−/−^ 2.5%; data not shown). Regardless of the treatment, all groups experienced weight gain throughout the testing period and were hence considered healthy.

#### 3.3.2 Elevated Zero Maze (EZM)

To assess genotype differences in anxiety‐related behaviour that might influence pain sensitivity, we tested all rats on the EZM prior to any intervention (data not shown). Two ♂WT rats and one ♂TRPV1^−/−^ rat were excluded from analysis due to falling from the EZM during the first 50% of the testing time. ♂TRPV1^−/−^ rats spent 38% more time in the open zones of the EZM than WT (*p* < 0.01, *d* = 1.5). Moreover, ♂TRPV1^−/−^ rats made five times more complete crossings (= transitions) of the open zones than their WT littermates (*p* < 0.01, *d* = 1.7). ♀TRPV1^−/−^ rats also exhibited less anxiety‐related behaviour than ♂WT rats (48% more time in open arms, 3.6 times more crossings).

### 3.4 Local Hypersensitivity, Latent and Manifest Sensitization After Repeated NGF Injections

**FIGURE 4:**
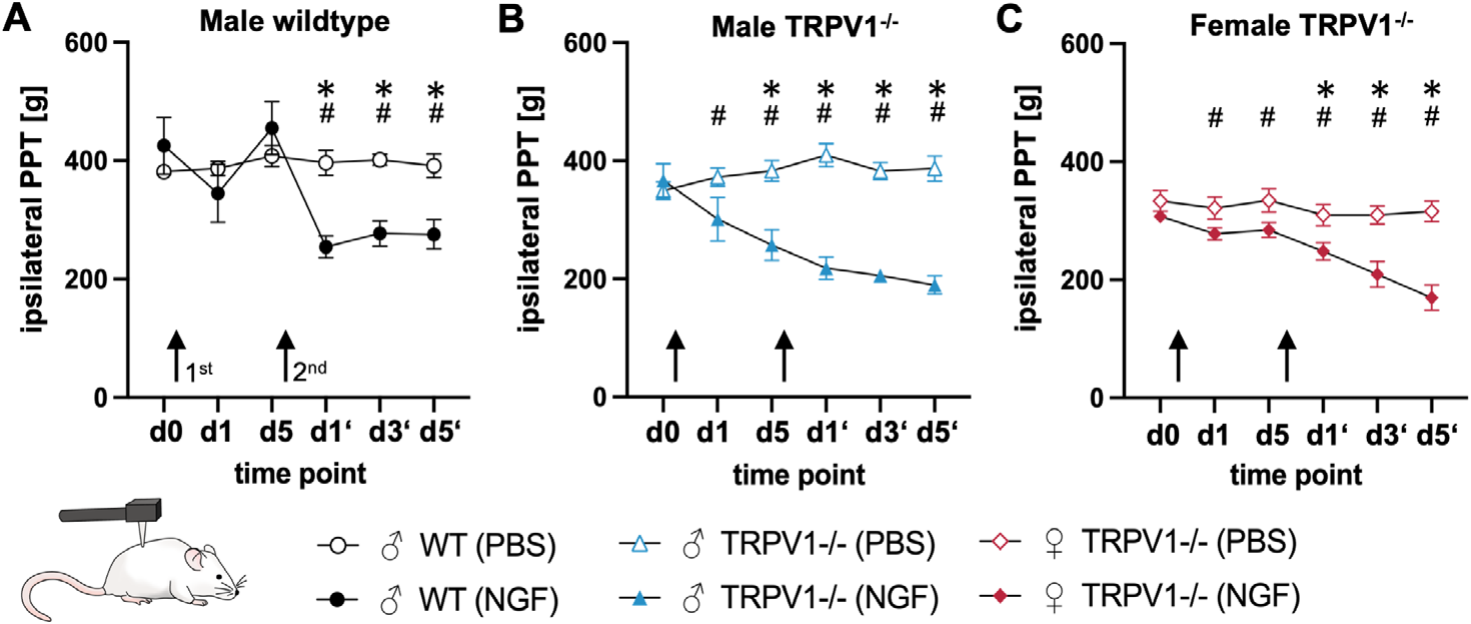
Repeated NGF injections induce manifest mechanical lumbar muscle hypersensitivity. Absolute local PPT values. *Y*‐axis shows pressure pain thresholds over lumbar muscles. Arrows indicate NGF/PBS injections (d0, d5). Significant time × treatment interaction (2‐way repeated measures ANOVA) was observed in male WT, male TRPV1^−/−^, and female TRPV1^−/−^. Post hoc comparisons (NGF vs. PBS) after correction for multiple comparisons are marked, *p < 0.05. Mean ± SEM; *n* = 5 each, # strong effect, Cohen's *d* ≥ 1. Time points shown are day 1 after the first injection (d1), day 5 after the first injection (d5), day 1 after the second injection (d1′), day 3 after the second injection (d3′) and day 5 after the second injection (d5′).

To assess local deep muscle hypersensitivity, we measured PPT of the MF muscles (Figure 4, Table 2). As shown in the figure, no significant differences were detected between NGF and control groups at day 1 (♂WT: *p* = 0.41; ♂TRPV1^−/−^: *p* = 0.60, Figure 4A,B). However, the between‐group comparison at this time point already showed a moderate effect size in ♂WT rats, indicating a trend toward lower PPT after NGF injection despite the lack of statistical significance. Analysis of the same dataset revealed that PPT was significantly reduced from baseline in both ♂WT (*p* < 0.01, *d* = 3.1) and ♂TRPV1^−/−^ (*p* = 0.01, *d* = 1.6), indicating a transient within‐group hypersensitivity. These within‐group comparisons over time are not displayed in the figure, which only depicts between‐group comparisons at each timepoint, but are reported here to highlight the temporal changes in hypersensitivity. The threshold normalized by d5 in ♂WT rats but remained low in ♂TRPV1^−/−^ rats. A second NGF injection caused further PPT decreases in both groups. ♀TRPV1^−/−^ rats also exhibited a decrease in PPT with lack of recovery after the first NGF injection and a prolonged hypersensitivity after the second NGF injection (Figure 4C).

In WT rats, a single NGF injection triggers initial hypersensitivity, followed by a remission to baseline PPT by day 5, marking a latent sensitization phase. A second NGF injection reactivates prolonged hypersensitivity, indicating manifest sensitization. TRPV1^−/−^ rats of both sexes lack this remission.

### 3.5 Remote Mechanical Hypersensitivity After Repeated Lumbar Intramuscular NGF Injections

**FIGURE 5:**
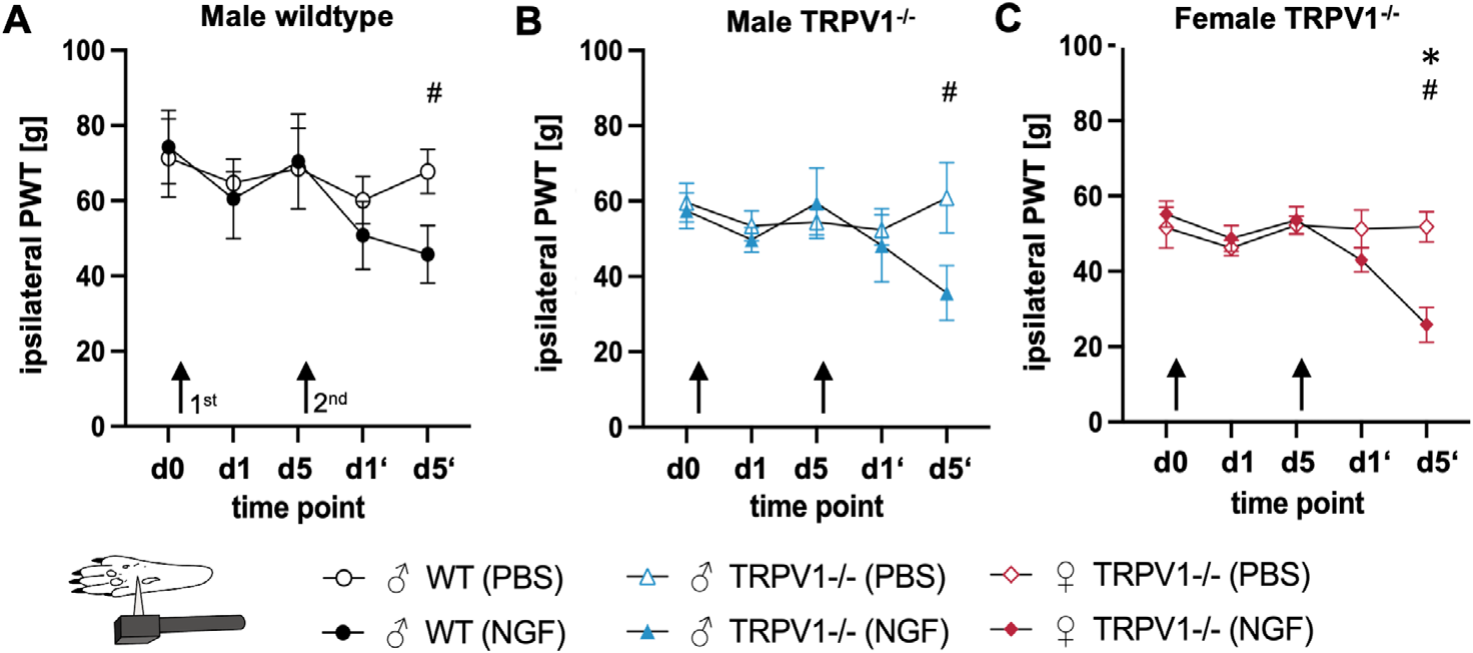
Repeated NGF injections induce remote mechanical hypersensitivity of the hind paws. Mechanical paw withdrawal thresholds (PWT) in ipsilateral hind paws decreased after two NGF injections in all groups, with strong effect sizes (# strong effect, Cohen's *d* ≥ 1); statistical significance was observed only in females; two‐way ANOVA, *p < 0.05; Mean ± SEM; *n* = 5 per group. Time points shown are day 1 after the first injection (d1), day 5 after the first injection (d5), day 1 after the second injection (d1′), and day 5 after the second injection (d5′).

We assessed mechanical hypersensitivity at the ipsilateral hind paws to evaluate spread of hypersensitivity to areas remote from the NGF injections (Figure 5, Table 2). There were non‐significant but notable decreases in PWT with large effect sizes at day 10 compared to baseline in all three groups: in ♂WT (*p* = 0.06, *d* = 1.5, Figure 5A), ♂TRPV1^−/−^ rats (*p* = 0.07, *d* = 1.3, Figure 5B), and ♀TRPV1^−/−^ rats (*p* < 0.01, *d* = 2.7, Figure 5C). No significant changes of PWT over time after PBS injections were observed in any group.

All NGF‐treated animals displayed significant remote mechanical hypersensitivity at the hind paw ipsilateral to the injection site by day 10, suggesting remote hypersensitivity. This spread had a delayed onset compared to the initial local hypersensitivity detected in ipsilateral PPT measurements. There was no genotype‐by‐treatment interaction, indicating that genotype did not affect treatment outcomes and no difference between sexes.

### 3.6 Changes in Exploratory Behaviour After Repeated Intramuscular NGF Injections

**FIGURE 6:**
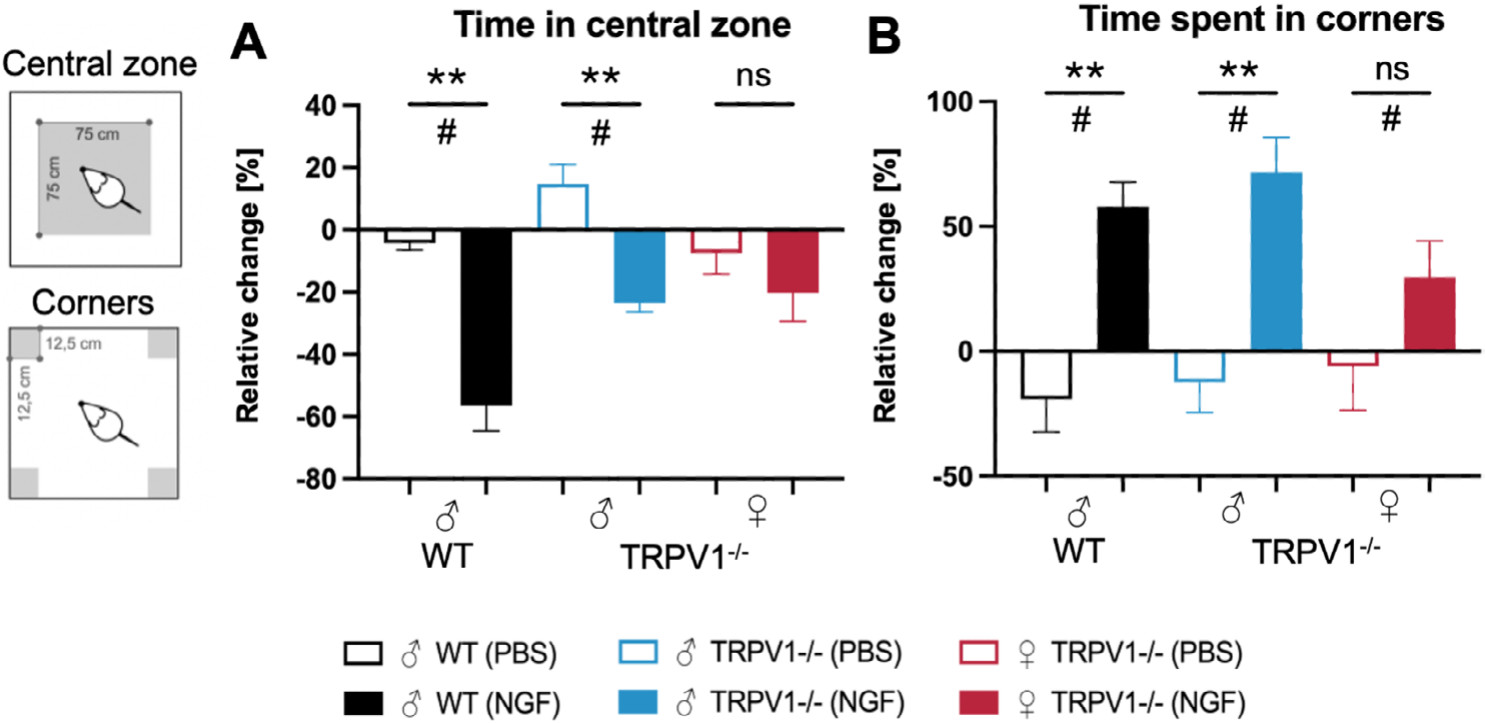
Repeated intramuscular NGF injections reduce exploratory behaviour. (A) Time spent in central zone of open field arena. (B) Time spent in corners of open field arena. Relative change (%) = (d2′–d‐1)/d‐1 × 100. Mean ± SEM; NGF vs. PBS comparison in all three groups: *T*‐tests for normal data, Mann–Whitney *U* test for non‐normal data (*n* = 5 each). ns = not significant, *p* ≥ 0.05; * *p* < 0.05; **p < 0.01; # strong effect, Cohen's *d* ≥ 1. Time points shown are day 1 after the first injection (d1), day 5 after the first injection (d5), day 1 after the second injection (d1′), and day 5 after the second injection (d5′).

To assess NGF's effects on anxiety‐like behaviour, exploratory behaviour in the open field arena was analysed before the first injection (d‐1) and 2 days after the second injection (d2′). Both NGF‐injected ♂WT and ♂TRPV1^−/−^ rats spent significantly less time in the central zone but more time in the corners compared with PBS treated animals (central zone: WT: *p* < 0.01, *d* = 4.0, ♂TRPV1^−/−^: *p* < 0.01, d = 3.5; corners: WT: *p* < 0.01, *d* = 2.9, ♂TRPV1^−/−^: *p* < 0.01, *d* = 2.9; Figure 6A,B). Anxiety‐like behaviour in ♀TRPV1^−/−^ rats was reflected by a non‐significant but medium effect‐size reduction in time spent in central zone (*p* = 0.30, *d* = 0.68) and a large, though non‐significant, increase in time spent in corners (*p* = 0.16, *d* = 1.01).

Two NGF injections increased thigmotaxis, indicating anxiety‐like behaviour across all three groups, with larger effects in males than in females.

### 3.7 Summary of Sex Differences Between Male and Female TRPV1 ^−/−^ Rats

Male and female TRPV1^−/−^ rats showed largely similar responses to NGF. The main difference was timing: males showed local PPT decreases after one NGF injection, while ♀TRPV1^−/−^ rats required two injections (d5′: *p* < 0.01, *d* = 2.5, Figure 4C). ANOVA confirmed a significant sex × treatment interaction on day 5 (*F*(1,4) = 16.0, *p* = 0.02), indicating earlier male responses. By d5′ this interaction was no longer significant (*F*(1,4) = 2.32, *p* = 0.2). ♀TRPV1^−/−^ rats also showed remote mechanical hypersensitivity at the hind paw (PWT d5′: *p* < 0.01, *d* = 2.6, Figure 5C).

## 4 Discussion

The NGF‐induced cpLBP model in WT rats led to a transient TRPV1 upregulation in the superficial spinal dorsal horn after a single NGF injection and to a stronger and longer lasting upregulation after a second NGF injection 5 days later. This pattern was consistent with the transition from latent to manifest sensitization as established in previous behavioural and electrophysiological studies in our and other laboratories (Hoheisel et al. 2013; Li et al. 2024; Reed et al. 2022). WT rats showed the expected temporal pattern of hypersensitivity in the NGF model (Hoheisel et al. 2013; Reed et al. 2022; Syrett et al. 2022): initial hypersensitivity with reduced ipsilateral PPT values, followed by an apparent remission. However, the re‐expression of hypersensitivity after a second NGF injection indicates a latent sensitization state rather than true recovery. In contrast, TRPV1^−/−^ rats did not enter this remission phase; PPT values remained low without returning to normal within 5 days after the initial NGF injection, and a second injection slightly decreased PPT further in both sexes. Thus, contrary to our hypothesis, NGF‐induced hypersensitivity was not reduced. Instead, these data suggest that the upregulation of TRPV1 may be involved in homeostatic maintenance rather than induction of spinal central sensitization.

### 4.1 NGF‐Induced Hypersensitivity Is Independent of TRPV1

We observed that the genetic removal of TRPV1 does not prevent the development of mechanical hypersensitivity in a non‐inflammatory model of myofascial pain. Both WT and TRPV1^−/−^ rats displayed reduced PPT in the lumbar back muscles ipsilateral to the injection site following NGF injections, indicating mechanical hypersensitivity independent of TRPV1. This outcome aligns with a prior study in TRPV1^−/−^ mice, where TRPV1 deletion did not affect mechanical hypersensitivity in an inflammation‐induced chronic muscle hyperalgesia model (Walder et al. 2012). However, it contrasts with another study in TRPV1^−/−^ mice where a single NGF injection was used to mimic acute post‐exercise pain (Ota et al. 2013). These differences may arise from timing differences related to chronic vs. acute pain models.

Female TRPV1^−/−^ rats exhibited NGF‐induced mechanical hypersensitivity comparable to males, with differences primarily in timing, similar to previous findings (Syrett et al. 2022). Females showed a delayed onset of hypersensitivity, but increased thigmotaxis was similar to males. This sex‐dependent temporal difference parallels findings from stress‐based pain models, where females showed latent and males immediate sensitisation (Singhal et al. 2025). Our data indicate that TRPV1 is not essential for the development of deep tissue hyperalgesia. Both sexes of TRPV1^−/−^ rats develop strong NGF‐induced hypersensitivity with minor timing differences.

### 4.2 Spinal Sensitization Occurs in Both WT and TRPV1 ^−/−^ Rats

In addition to local hypersensitivity at the injected low back muscle, we also observed signs of hypersensitivity in remote areas, where mechanical hypersensitivity was indicated by reduced PWT values in both WT and TRPV1^−/−^ rats. This occurred in the hind paws' L4 dermatome, distinct from the injected muscle's L2 spinal segment projection, suggesting central sensitization. Hoheisel et al. (2013) demonstrated that repeated NGF input activates previously silent synaptic connections, forming new receptive fields (Hoheisel et al. 2013). These connections predominantly involved deep tissues (e.g., muscle, fascia, ligaments), while PWT testing targets skin hypersensitivity. Previous research has yielded mixed results regarding the impact of lumbar NGF injections on mechanical hind paw thresholds. Bilateral intramuscular NGF injections in mice led to reduced PWT (La Porta and Tappe‐Theodor 2020). Meanwhile, Reed et al. (2020) observed decreased PWT after unilateral NGF injections only in female rats (Reed et al. 2020), with two other studies (Reed et al. 2022; Syrett et al. 2022) showing no change in PWT in both male and female rats. Bilaterally reduced PWT has been linked to stress and the dysfunction of the diffuse noxious inhibitory system (La Porta and Tappe‐Theodor 2020). A recent study proposed that remote hypersensitivity could be due to referred pain, explained by the convergence projection theory, where sensory inputs from the low back and hindpaw converge on sensitized spinal dorsal horn neurons (Li et al. 2024). Such mechanisms are likely involved in the transition from localized cpLBP to chronic widespread pain. NGF injections lowered hind paw withdrawal thresholds in both WT and TRPV1^−/−^ rats, indicating TRPV1‐independent central sensitization with remote hypersensitivity.

Two related mechanisms have been described: hyperalgesic priming and latent sensitization (Price and Ray 2019). While both involve priming and engage peripheral and central processes, latent sensitization is distinguished by CNS‐driven inhibitory mechanisms that suppress nociceptive hypersensitivity, allowing its rapid re‐emergence when inhibition is removed. Although our approach does not follow the classical pharmacological definition, our findings demonstrating a critical role of spinal mechanisms in the NGF model are consistent with the concept of latent sensitization.

### 4.3 NGF Alters Exploratory Behaviour

Beyond sensory thresholds, NGF injections also altered exploratory behaviour. Both WT and TRPV1^−/−^ rats of both sexes showed increased thigmotaxis, spending more time in the corners and periphery of the open field and travelling less overall. Thigmotaxis is commonly interpreted as a marker of anxiety, which is also observed in chronic pain conditions. However, in the context of our low back pain model, reduced locomotion may also reflect movement‐evoked pain, which would naturally discourage the animals from crossing the open center of the field. Based on the present data alone, it is not possible to distinguish clearly between anxiety‐related behaviour and pain‐related avoidance, and both mechanisms may contribute. NGF injections reduce exploration and increase thigmotaxis, probably reflecting a combination of anxiety‐like behaviour and movement‐evoked pain.

### 4.4 NGF Enhances TRPV1 Expression in the Superficial Spinal Dorsal Horn

In WT rats, we observed upregulation of TRPV1 after two intramuscular injections of NGF. These findings are in line with previous studies showing enhanced TRPV1 expression in the peripheral nerve endings and DRGs (but not the spinal cord) following single NGF applications via different routes, such as intraplantar and intraperitoneal injections (Eskander et al. 2015; Ji et al. 2002). The neuronal basis of NGF‐induced cpLBP is still unknown. Human nucleus pulposus cells were shown to secrete large amounts of NGF, for example, in response to bacterial infection via activation of TLR‐2 receptor and NF‐kB p65/JNK or ROS‐related pathways (Jiao et al. 2022). NGF may activate tropomyosin receptor kinase A and p75 and thereby induce sensitization in spinal cord and upregulation of TRPV1 (Chen et al. 2024; Jiao et al. 2022; Reed et al. 2022). Our study supports the prediction of an upregulation of spinal TRPV1 after (repeated) muscle NGF, but contradicts TRPV1's proposed role in initiation of cpLBP. Clinical studies had found that long‐term application of topical capsaicin slightly reduced LBP (Cashin et al. 2025), this effect may be explained by downregulation of peripheral not spinal TRPV1. Our study extends this body of knowledge by showing TRPV1 upregulation in the superficial spinal dorsal horn, which is an important site in central sensitization, and by showing its facilitation by nociceptive priming in the latent sensitization model of cpLBP. However, this upregulation did not correspond with behavioural responses to lumbar muscle stimulation. This dissociation suggests that other mechanisms may drive initiation of latent hypersensitivity, while TRPV1 may contribute instead to homeostatic maintenance of nociceptive system responsivity.

### 4.5 TRPV1 Contributes to Homeostatic Plasticity

Some previous studies have also reported antinociceptive effects of TRPV1 in mechanical hypersensitivity. For instance, Walder et al. (2012) demonstrated that male TRPV1^−/−^ mice showed increased mechanical hypersensitivity to repeated mechanical stimulation, suggesting that TRPV1 may have inhibitory effects on the development of mechanical hypersensitivity (Walder et al. 2012). Bölcskei et al. (2005) also showed that male TRPV1^−/−^ mice developed higher mechanical hypersensitivity in non‐traumatic chronic pain models, proposing that TRPV1 may be involved in neuroendocrine functions, including the release of somatostatin (Bölcskei et al. 2005). While the exact mechanisms underlying the observed antinociceptive effects of TRPV1 in mechanical hypersensitivity remain to be elucidated, they suggest a role of TRPV1 in homeostatic plasticity in the spinal cord. Current research supports the involvement of TRPV1 in homeostatic plasticity within other parts of the CNS such as the hippocampus (McGrady et al. 2020)—including induction of both, long‐term potentiation, LTP (Yang et al. 2014), *and* long‐term depression, LTD (Kano 2014), as shown before for example, within nucleus accumbens (Grueter et al. 2010). Its ability to regulate intracellular calcium levels and influence synaptic strength positions TRPV1 as a modulator in maintaining neural circuit stability, especially under conditions of stress or injury. Previous studies connected TRPM3‐mediated calcium signalling, mitochondrial dynamics, and enhanced synaptic activity as underlying mechanisms in NGF‐induced spinal sensitization (Wang et al. 2022, 2023). TRPV1 knockout may shift reliance to TRPM3‐mediated pathways, which sustain manifest sensitization through calcium influx, mitochondrial activity, and synaptic transmission, highlighting distinct yet redundant roles of TRPV1 and TRPM3 in NGF‐induced sensitization. TRPV1 likely modulates spinal homeostasis and thereby limits progression to chronic sensitization.

In conclusion, our findings suggest that TRPV1 is upregulated in, but not essential for, NGF‐induced spinal sensitization leading to local or remote mechanical hypersensitivity. Instead, TRPV1 appears to maintain homeostatic spinal plasticity by enabling the remission phase after a single NGF injection, allowing the nervous system to stabilize in a sensitized but behaviorally silent state characteristic of latent sensitization. In the absence of TRPV1, this homeostatic control was lost, and hypersensitivity progressed directly to the prolonged state of manifest sensitization. These results highlight TRPV1 as a potential regulator of spinal homeostasis in the context of chronic musculoskeletal pain also displaying beneficial effects against chronic manifestation of LBP.

## Funding

This study received financial support by the German Research Foundation, DFG grants IRTG 1874 (International Research Training Group on Diabetic Microvascular Complications DIAMICOM to WG, RDT), GRK2350 (Research Training Grant on The Impact of Adverse Childhood Experiences on Psychosocial and Somatic Conditions Across the Lifespan to RDT), and TR 236/24‐1 (individual grant to RDT). We are grateful for the support provided by scholarships of the Medical Faculty Mannheim (to VB, JRH).

## Conflicts of Interest

The authors declare no conflicts of interest.

## Supporting information


**Figure S1:** Mechanical pain thresholds of the lumbar muscle and hind paw contralateral to injection site.

**Figure S2:** Spinal TRPV1 immunofluorescence contralateral to injection site.

**Table S1:** Statistical analysis of contralateral PPT.

**Table S2:** Statistical analysis of contralateral PWT.
