# Supplementary material for: Bidirectional Roles of TRPV1 in a Latent Sensitization Model of Myofascial Low Back Pain
Source: Eur J Pain. 2026 Mar 26;30(4):e70255. doi: 10.1002/ejp.70255 (PMC13019274; doi:10.1002/ejp.70255)
Supplement: Supplementary file 1 — Figure S1: Mechanical pain thresholds of the lumbar muscle and hind paw contralateral to injection site. [file EJP-30-0-s001.docx]

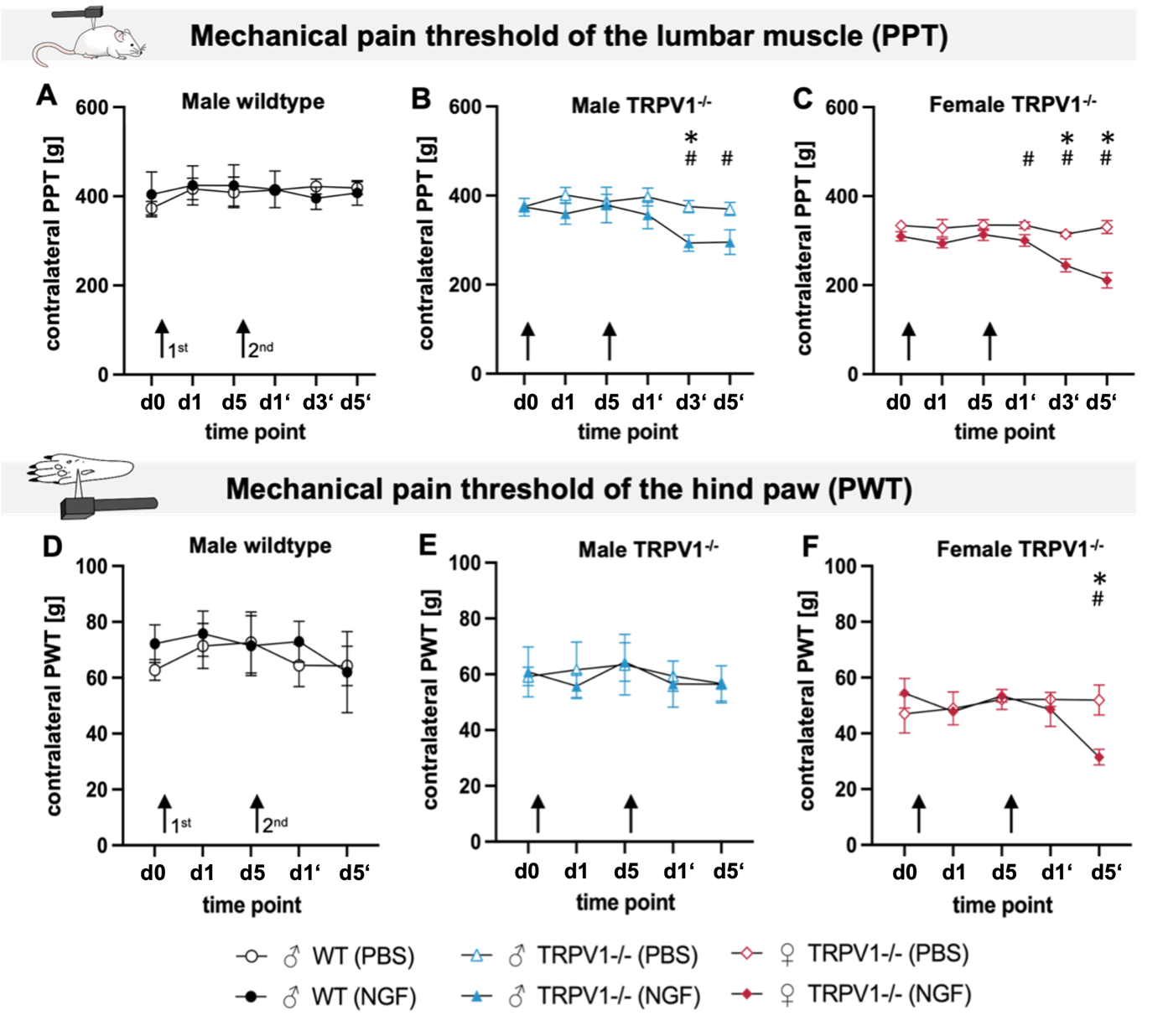


**Fig. S1 Mechanical pain thresholds of the lumbar muscle and hind paw contralateral to injection site. A-C** **PPT contralateral**. Two NGF injections reduced PPT only in TRPV1-/- rats, with strong effect sizes (# strong effect, Cohen's d ≥ 1) and statistical significance (* p < 0.05, two-way ANOVA). Mean ± SEM; n = 5 each group. **D-F** **PWT contralateral**. Significant reduction on d10 was observed only in female TRPV1^-/-^ rats. Mean ± SEM; n = 5 each, two-way ANOVA. No significant differences between NGF and PBS treated animals were observed. * p < 0.05; # strong effect, Cohen's d ≥ 1. Time points shown are day 1 after the first injection (d1), day 5 after the first injection (d5), day 1 after the second injection (d1′), day 3 after the second injection (d3′) and day 5 after the second injection (d5′*).*

NGF, nerve growth factor; PBS, phosphate-buffered saline; PWT, paw withdrawal threshold; PPT, pressure pain threshold; SEM, standard error of mean; WT, wildtype.
