# Supplementary material for: Bidirectional Roles of TRPV1 in a Latent Sensitization Model of Myofascial Low Back Pain
Source: Eur J Pain. 2026 Mar 26;30(4):e70255. doi: 10.1002/ejp.70255 (PMC13019274; doi:10.1002/ejp.70255)
Supplement: Supplementary file 2 — Figure S2: Spinal TRPV1 immunofluorescence contralateral to injection site. [file EJP-30-0-s004.docx]

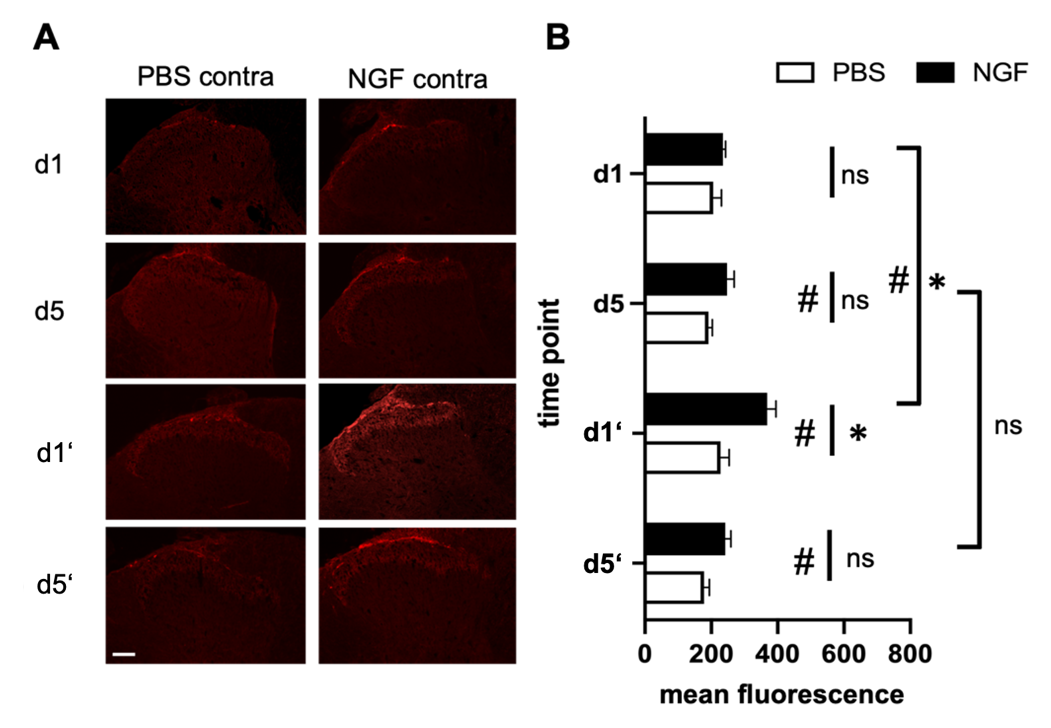


**Fig. S2 Spinal TRPV1 immunofluorescence contralateral to injection site. A** Representative image of the mean fluorescence intensity of TRPV1 in the ipsilateral dorsal horn of the lumbar spinal cord segment L2. Images were acquired under identical conditions, with parameters optimized for NGF-treated tissue; thus, basal TRPV1 expression in PBS controls may appear faint. **B** TRPV1 is time-dependently upregulated in spinal dorsal horn after NGF injection (16 bit, scale: 0-65536). Mean ± SEM, n = 5 each, two-way ANOVA. ns = not significant, p ≥ 0.05; * p < 0.05; # strong effect, Cohen's d ≥ 1. Scale bar: 200 µm. Time points shown are day 1 after the first injection (d1), day 5 after the first injection (d5), day 1 after the second injection (d1′), and day 5 after the second injection (d5′*).*

NGF, nerve growth factor; PBS, phosphate-buffered saline; WT, wildtype.
