# Supplementary material for: Bidirectional Roles of TRPV1 in a Latent Sensitization Model of Myofascial Low Back Pain
Source: Eur J Pain. 2026 Mar 26;30(4):e70255. doi: 10.1002/ejp.70255 (PMC13019274; doi:10.1002/ejp.70255)
Supplement: Supplementary file 3 — Table S1: Statistical analysis of contralateral PPT. [file EJP-30-0-s002.docx]

|  | **♂ WT: PBS vs. NGF** | | **♂ TRPV1-/-: PBS vs. NGF** | | **♀ TRPV1-/-: PBS vs. NGF** | |
| --- | --- | --- | --- | --- | --- | --- |
| **timepoint** | **P value** | **Cohen's d** | **P value** | **Cohen's d** | **P value** | **Cohen's d** |
| d0 | 0,584 | 0,37 | 0,959 | 0,03 | 0,162 | 0,98 |
| d1 | 0,881 | 0,10 | 0,189 | 0,64 | 0,168 | 0,99 |
| d5 | 0,796 | 0,18 | 0,866 | 0,11 | 0,264 | 0,76 |
| d1‘ | 0,969 | 0,03 | 0,317 | 0,68 | 0,062 | 1,44 |
| d3‘ | 0,409 | 0,58 | 0,008 | 2,29 | 0,006 | 2,93 |
| d5‘ | 0,742 | 0,22 | 0,055 | 1,50 | 0,001 | 3,36 |

**Tab. S1 Statistical analysis of contralateral PPT.** Repeated measures ANOVA compared pressure pain thresholds in NGF- and PBS-treated animals across time points, using Geisser-Greenhouse correction. Normality (Shapiro-Wilk) and homogeneity of variance (Levene’s) were tested beforehand. Since assumptions were met, two-way ANOVA with Tukey’s post hoc for multiple comparisons was used. n = 5 each. Cohen’s d was calculated as the mean difference divided by the pooled standard deviation. Time points shown are day 1 after the first injection (d1), day 5 after the first injection (d5), day 1 after the second injection (d1′), day 3 after the second injection (d3′) and day 5 after the second injection (d5′*).* NGF, nerve growth factor; PBS, phosphate-buffered saline; PPT, pressure pain threshold; WT, wildtype.
