# Supplementary material for: Bidirectional Roles of TRPV1 in a Latent Sensitization Model of Myofascial Low Back Pain
Source: Eur J Pain. 2026 Mar 26;30(4):e70255. doi: 10.1002/ejp.70255 (PMC13019274; doi:10.1002/ejp.70255)
Supplement: Supplementary file 4 — Table S2: Statistical analysis of contralateral PWT. [file EJP-30-0-s003.docx]

|  | **♂ WT: PBS vs. NGF** | | **♂ TRPV1-/-: PBS vs. NGF** | | **♀ TRPV1-/-: PBS vs. NGF** | |
| --- | --- | --- | --- | --- | --- | --- |
| **timepoint** | **P value** | **Cohen's d** | **P value** | **Cohen's d** | **P value** | **Cohen's d** |
| d0 | 0,269 | 0,79 | 0,866 | 0,11 | 0,414 | 0,55 |
| d1 | 0,711 | 0,26 | 0,616 | 0,34 | 0,866 | 0,11 |
| d5 | 0,943 | 0,05 | 0,942 | 0,05 | 0,764 | 0,20 |
| d1‘ | 0,448 | 0,54 | 0,779 | 0,18 | 0,607 | 0,35 |
| d5‘ | 0,895 | 0,09 | 0,974 | 0,02 | 0,015 | 2,13 |

**Tab. S2 Statistical analysis of contralateral PWT.** Repeated measures ANOVA compared paw withdrawal thresholds in NGF- and PBS-treated animals across time points, using Geisser-Greenhouse correction. Normality (Shapiro-Wilk) and homogeneity of variance (Levene’s) were tested beforehand. Since assumptions were met, two-way ANOVA with Tukey’s post hoc for multiple comparisons was used. n = 5 each. Cohen’s d was calculated as the mean difference divided by the pooled standard deviation. Time points shown are day 1 after the first injection (d1), day 5 after the first injection (d5), day 1 after the second injection (d1′), and day 5 after the second injection (d5′*).* NGF, nerve growth factor; PBS, phosphate-buffered saline; PWT, paw withdrawal threshold; WT, wildtype.
